# Prediction of corneal power vectors after cataract surgery with toric lens implantation–A vector analysis

**DOI:** 10.1371/journal.pone.0288316

**Published:** 2023-09-08

**Authors:** Achim Langenbucher, Nóra Szentmáry, Alan Cayless, Jascha Wendelstein, Peter Hoffmann

**Affiliations:** 1 Department of Experimental Ophthalmology, Saarland University, Homburg/Saar, Germany; 2 Dr. Rolf M. Schwiete Center for Limbal Stem Cell and Aniridia Research, Saarland University, Homburg/Saar, Germany; 3 Department of Ophthalmology, Semmelweis-University, Budapest, Hungary; 4 School of Physical Sciences, The Open University, Milton Keynes, United Kingdom; 5 Department of Ophthalmology, Johannes Kepler University Linz, Linz, Austria; 6 Augen- und Laserklinik Castrop-Rauxel, Castrop-Rauxel, Germany; Chiemsee Augen Tagesklinik, Technical University of Munich, GERMANY

## Abstract

**Background:**

Intraocular lenses are typically calculated based on a pseudophakic eye model, and for toric lenses (tIOL) a good estimate of corneal astigmatism after cataract surgery is required in addition to the equivalent corneal power. The purpose of this study was to investigate the differences between the preoperative IOLMaster (IOLM) and the preoperative and postoperative Casia2 (CASIA) tomographic measurements of corneal power in a cataractous population with tIOL implantation, and to predict total power (TP) from the IOLM and CASIA keratometric measurements.

**Methods:**

The analysis was based on a dataset of 88 eyes of 88 patients from 1 clinical centre before and after tIOL implantation. All IOLM and CASIA keratometric and total corneal power measurements were converted to power vector components, and the differences between preoperative IOLM or CASIA and postoperative CASIA measurements were assessed. Feedforward neural network and multivariate linear regression prediction algorithms were implemented to predict the postoperative total corneal power (as a reference for tIOL calculation) from the preoperative IOLM and CASIA keratometric measurements.

**Results:**

On average, the preoperative IOLM keratometric / total corneal power under- / overestimates the postoperative CASIA keratometric / real corneal power by 0.12 dpt / 0.21 dpt. The prediction of postoperative CASIA real power from preoperative IOLM or CASIA keratometry shows that postoperative total corneal power is systematically (0.18 dpt / 0.27 dpt) shifted towards astigmatism against the rule, which is not reflected by keratometry. The correlation of postoperative CASIA real power to the corresponding preoperative CASIA values is better than those as compared to the preoperative IOLM keratometry. However, there is a large variation from preoperative IOLM or CASIA keratometry to the postoperative CASIA real power of up to 1.1 dpt (95% confidence interval).

**Conclusion:**

One of the challenges of tIOL calculation is the prediction of postoperative total corneal power from preoperative keratometry. Keratometric power restricted to a front surface measurement does not fully reflect the situation of corneal back surface astigmatism, which typically adds some extra against the rule astigmatism.

## Background

Toric lens (tIOL) implantation is a popular option for correction of corneal astigmatism during cataract surgery. Indications for toric lenses vary between surgical centres and countries, but in general, as toric lenses are available from all intraocular lens (IOL) manufacturers starting with a cylindrical power of around 1 dpt, such tIOLs are used for corneal astigmatism values of around 0.75 dpt or higher [1–3].

In general, calculation of tIOLs could be performed using empirical concepts (based on statistical models), paraxial formulae (based on a model eye) or raytracing. With paraxial formulae a simple pseudophakic model eye with 3–5 refracting surfaces is defined including a spherical or spherocylindrical spectacle refraction (target refraction), a thin or thick lens cornea with 1 or 2 spherocylindrical surfaces, and a thin or thick toric implant. In most cases the exact design of the tIOL is not known, and therefore calculations are simplified to using a thin lens based on the manufacturer’s label data. With (full aperture) raytracing we also use a pseudophakic model eye, but here the shape of all refracting surfaces (spectacle correction, corneal front and back surface, and tIOL front and back surface), the alignment of all surfaces relative to the visual axis, and the outline of the aperture has to be known. In reality, many assumptions and simplifications are made for raytracing calculation of tIOLs: e.g. in the case of non-plano target refraction the spectacle correction is assumed to be represented by a thin sphero(cylindric) lens, and all refracting surfaces and the (circular) aperture stop are assumed to be coaxially aligned.

In most cases tIOLs are calculated based on measurement data of the corneal front surface as derived from a manual keratometer, automated keratometer integrated in a biometer, or a corneal topographer. Even where corneal back surface data are available from a Scheimpflug [4] or optical coherence tomographer [5–7], many calculation schemes for tIOLs use keratometric data. However, it is well known in ophthalmology that a keratometer or the simulated keratometry SimK of a topographer does not fully describe the corneal power [8–10]. As the measurement is restricted to the corneal front surface radii (derived in the mid periphery of the cornea), assumptions regarding the corneal thickness and the corneal back surface curvature are required in order to provide the power of the entire cornea [8]. In addition to the assumptions of the corneal back surface curvature, the ratio of the corneal front to back surface radii may vary for both cardinal corneal meridians, and the cardinal meridians of both corneal surfaces may be misaligned. Beside direct measurement of the curvature of both corneal surfaces, several statistical models have been published (and used in several tIOL calculation software modules) to estimate the portion of corneal back surface astigmatism which is not properly described by a keratometer [9]. However, such statistical corrections models are in fact limited to describing the systematic portion of the deviation of the real corneal power from the keratometric power, and stochastic variations cannot be mapped [8, 9]. In addition to the variations of corneal power resulting from the corneal back surface shape, the calculation is based on a pseudophakic instead of the phakic model eye, and we have to consider the potential change of corneal front and back surface curvature as a result of cataract surgery.

The standard notation of corneal power with base curve radius, astigmatism, and orientation of the astigmatism is not really helpful for describing differences between corneal power data or the change from the preoperative to the postoperative situation [11, 12]. Instead, this standard notation is typically transformed to a component notation where the power vector contains the equivalent power together with the projection of the astigmatism to the 0°/90° and to the 45°/135° meridian [11–14]. In contrast to the standard notation, the components of the power vector can be directly added or subtracted to consider a refractive surface or the change due to surgery [11, 12].

The **purpose of this study** was

- to assess the keratometric, anterior and posterior surface and total corneal power derived from measurement of a patient cohort before and after cataract surgery with implantation of a toric intraocular lens,
- to analyse the changes of the power vectors from the preoperative to the postoperative situation,
- to derive a shallow feedforward neural network model and a multilinear regression model for predicting the postoperative corneal power to be used for toric lens power calculation from the preoperative corneal front surface data.

## Methods

### Dataset for our study and surgical details

In total, a dataset including 211 eyes measured with the IOLMaster 700 (IOLM, Zeiss, Jena, Germany) and the Casia2 anterior segment OCT (CASIA, Tomey, Nuremberg, Germany) from one clinical centre (Augen- und Laserklinik Castrop-Rauxel, Germany) was considered for this retrospective study. All cataractous eyes were treated with a toric intraocular lens implant (Vivinex toric, Hoya, Singapore). The eyes were measured before cataract surgery with the IOLMaster biometer and the Casia2 tomographer and 6–12 weeks postoperatively with the Casia 2 tomographer. In addition, the power and orientation of the implanted lens, the eye (OS or OD) and the patient age were recorded. At the postoperative follow-up, in addition to the Casia2 examination the refraction was measured by an experienced optometrist manually using trial glasses in a trial frame. All data were anonymised at source and stored in a.CSV file, which was transferred to us for further analysis. In cases where both eyes of a patient were included, one eye was chosen at random and the other was excluded. Data tables were reduced to the relevant parameters required for our analysis, consisting of:

IOLMaster: axial length (AL in mm), central corneal thickness (CCT in mm), anterior chamber depth (ACD in mm) measured from the corneal epithelium to the front apex of the crystalline lens, central thickness of the crystalline lens (LT in mm), corneal front surface curvature (flat meridian IOLMR1a in mm with axis IOLMA1a in°; steep meridian IOLMR2a in mm with axis IOLMA2a in°), corneal back surface curvature (flat meridian IOLMR1p in mm with axis IOLMA1p in°; steep meridian IOLMR2p in mm with axis IOLMA2p in°), total keratometry based on corneal front and back surface curvature (flat meridian IOLMR1t in mm with axis IOLMA1t in°; steep meridian IOLMR2t in mm with axis IOLMA2t in°);

CASIA: corneal front surface curvature (flat meridian CASIAR1a in mm with axis CASIAA1a in°; steep meridian CASIAR2a in mm with axis CASIAA2a in°), corneal back surface curvature (flat meridian CASIAR1p in mm with axis CASIAA1p in°; steep meridian CASIAR2p in mm with axis CASIAA2p in°), real power based on corneal front and back surface curvature (flat meridian CASIAP1r in dpt with axis CASIAA1r in°; steep meridian CASIAP2r in dpt with axis CASIAA2r in°). The preoperative / postoperative measurements with the Casia2 were indexed with (.)_pre / (.)_post;

Missing data, or data with a ‘Failed’ or ‘Warning’ marker in the quality check for keratometry, total keratometry, back surface curvature, or AL, CCT, ACD, LT, provided by the IOLMaster 700 software were excluded. Incomplete data or data with a ‘Warning’ marker at the preoperative or postoperative measurement with the Casia2, and also measurements in mydriasis (pupil size > 5.5 mm) or with changes in the pupil size from preoperative to postoperative measurement of more than 1.5 mm, were also excluded. After checking for ‘Successful’ measurement, a dataset containing records of measurements from N = 88 eyes with preoperative and postoperative measurements was used.

All surgeries were performed under topical anaesthesia between January 2019 and June 2022 by 2 experienced surgeons. Before surgery, the steep meridian of the cornea indicated by optical biometry (tomography used for crosschecking) was marked using an adjustable pendulum marker (Geuder AG). Surgery was performed using coaxial microphaco instruments (Geuder AG) with a 2.2 mm incision width. All incisions were placed temporally (180° in right eyes, 0° in left eyes). The creation of a continuous curvilinear capsulorhexis slightly smaller than the IOL optic diameter (approximately 5.5 mm) was performed in all cases. After a standard phacoemulsification procedure, the Vivinex tIOL was inserted and aligned with its marker at the steep corneal meridian (CASIAA2r_pre), and the CCI and both paracenteses were hydrated. After the ophthalmic viscosurgical device was thoroughly removed by irrigation/aspiration, final positioning was performed with the aid of the infusion handpiece and a Sinskey hook. The correct position was checked intraoperatively by retinoscopy.

The data were transferred to Matlab (Matlab 2021a, MathWorks, Natick, USA) for further processing. The local ethics committee provided a waiver for this study (Ärztekammer des Saarlandes, 157/21).

### Preprocessing of the data

Custom software was written in Matlab to decompose the refractive power of keratometry, total keratometry and back surface power from standard notation (corneal curvature in both cardinal meridians with axis orientations), the postoperative refraction at the spectacle plane (REFS at REFA and REFS+REFC at REFA+90°), and the thin lens model of the tIOL (IOLP1 = IOLP-0.5·IOLT at IOLA and IOLP2 = IOLP+0.5·IOLT at IOLA+90°) into power vector components in terms of (spherical) equivalent power (.)_EQ_, the astigmatism projected to the 0°/90° meridian (.)_C0_, and astigmatism projected to the 45°/135° meridian (.)_C45_. For the corneal front surface / back surface we used the refractive indices derived from the Liou-Brennan schematic model eye with n_2_-n_1_ = 0.376 / n_2_-n_1_ = -0.040 and for keratometry / total keratometry we used the Zeiss keratometer index (1.332) with n_2_-n_1_ = 0.332:

$$\begin{array}{ccc} {\left(.\right)}_{EQ} & = & 500∙(n_{2}-n_{1})(\frac{1}{(.)R2}+\frac{1}{(.)R1})\\ {\left(.\right)}_{C0} & = & (n_{2}-n_{1})(\frac{1}{(.)R2}-\frac{1}{(.)R1})∙\cos\left(\frac{\pi }{90}(.)A1\right)\\ {\left(.\right)}_{C45} & = & (n_{2}-n_{1})(\frac{1}{(.)R2}-\frac{1}{(.)R1})∙\sin\left(\frac{\pi }{90}(.)A1\right) \end{array}$$

For the real power of the CASIA and the power of the tIOL, which are already given in dpt instead of mm radius of curvature, the conversion to power vector components was performed using:

$$\begin{array}{ccc} {\left(.\right)}_{EQ} & = & 0.5∙((.)P1+(.)P2)\\ {\left(.\right)}_{C0} & = & ((.)P2-(.)P1)∙\cos\left(\frac{\pi }{90}(.)A1\right)\\ {\left(.\right)}_{C45} & = & ((.)P2-(.)P1)∙\sin\left(\frac{\pi }{90}(.)A1\right) \end{array}$$

Based on the assumption that left and right eyes behave symmetrically, the power vector components in 45°/135° anterior and posterior surface power, keratometry, total keratometry / real power, refraction, and the tIOL were reversed in sign for all left eyes in our dataset in order to present the data in the same orientation as for right eyes.

The differences between the CASIA and IOLM preoperative measurements and the change of corneal power from preoperative to postoperative (CASIA) was derived by subtracting the respective power vector components for keratometry, total keratometry / real power, and front and back surface.

### Setup of the prediction algorithm

A feedforward shallow multi-layer multi-output neural network was set up for predicting the postoperative real power vector components CASIAr_C0__post and CASIAr_C45__post from the following input parameters: corneal front surface power measured preoperatively with the IOLM (IOLMa_C0_ and IOLMa_C45_), or the preoperatively or postoperatively measured corneal front surface power from the Casia (CASIAa_C0__pre and CASIAa_C45__pre or CASIAa_C0__post and CASIAa_C45__post). For the training function we used the Levenberg-Marquardt algorithm, as this algorithm is known to exhibit a good performance in terms of convergence and stability. Optimisation was based on minimising the mean value of the squared prediction error (NNPE) derived from the 2 vector components of the measured (CASIAr_C0__post and CASIAr_C45__post) and the predicted (NNCASIAr_C0__post and NNCASIAr_C45__post) real power vector components:

$$NNPE=1/2∙(({CASIAr}_{C0}\_post-{NNCASIAr}_{C0}\_post{)}^{2}+({CASIAr}_{C45}\_post-{NNCASIAr}_{C45}\_post{)}^{2}).$$

We set up a shallow feedforward neural network with 2 hidden layers with 10 / 8 neurons for the 1^st^ and 2^nd^ hidden layer to keep the network and the structure of the perceptron simple.

In addition, we defined a multivariate linear regression model using the power vector components derived with the IOLM (IOLMa_C0_ and IOLMa_C45_), or the preoperatively or postoperatively measured corneal front surface power from the Casia (CASIAa_C0__pre and CASIAa_C45__pre or CASIAa_C0__post and CASIAa_C45__post) to predict the 2 power vector components vector components CASIAr_C0__post and CASIAr_C45__post as output parameters. Again, optimisation was performed in terms of minimising the mean value of the squared prediction error (REGPE) derived from the 2 vector components of the measured (CASIAr_C0__post and CASIAr_C45__post) and the predicted (REGCASIAr_C0__post and REGCASIAr_C45__post) real power vector components

$$REGPE=1/2∙(({CASIAr}_{C0}\_post-{REGCASIAr}_{C0}\_post{)}^{2}+({CASIAr}_{C45}\_post-{REGCASIAr}_{C45}\_post{)}^{2}).$$

For both prediction models (feedforward neural network and multivariate regression model) the dataset with N = 88 data points was split using a random selection into a training set (70%, N = 62), a validation set (N = 13) and a test set (N = 13). The neural network and the multivariate regression were both trained using the training dataset, and the neural network was back-projected using the validation dataset. The prediction performance was assessed using the N = 13 test dataset. In a final step both prediction strategies trained on the training data (N = 62) were applied to the entire dataset (N = 88) for graphical presentation of the predictions versus the measurements.

For graphical presentation of the results, double angle plots were used to display the power vector components C_0°_ (in the horizontal direction) and C_45°_ (in the vertical direction), as well as the differences between IOLM and CASIA and the changes of the power vector from preoperative to postoperative examination. The 90% confidence ellipse was derived from the power vector components and vector component changes/differences C_0°_ and C_45_ using eigenvalue decomposition. The respective error ellipses together with the respective centroids (centres of the error ellipses) and orientations of the ellipses were overlaid on the double angle plots.

## Results

In total, after filtering and applying inclusion/exclusion criteria to the measurements, our dataset consists of 88 eyes (39 left and 49 right eyes from 50 female and 38 male patients from one clinical centre). Biometric measures derived from the IOLM ranged between 24.1154±1.6258 mm (median 23.7459 mm; 95% confidence interval 21.9193 to 28.5735 mm) for AL, 0.5574±0.0368 mm (median 0.5541 mm; 95% confidence interval 0.4897 to 0.6261 mm) for CCT, 3.1882±0.3936 mm (median 3.1640 mm; 95% confidence interval 2.4595 to 3.9277 mm) for ACD, and 4.6684±0.5250 mm (median 4.6789 mm; 95% confidence interval 3.3552 to 5.5855 mm) for LT.

**Table 1** shows the explorative data of the power vector components for the preoperative biometric measurements made with the IOLM and the preoperative and postoperative measurements made with the anterior segment OCT CASIA. The power vector components are listed for the corneal front and back surface power, for the keratometry calculated with the Zeiss keratometer index, and for the total keratometry (IOLM) and the real power (CASIA). The power vector components in 45°/135° were reversed in sign for all left eyes in our dataset in order to present the data in the same orientation as for right eyes.

**Table 1:** Power vector components derived from the preoperative biometric measurement with the IOLMaster 700 (IOLM), and the preoperative and postoperative measurement with the Casia2 (CASIA). Decompositions into power vectors were performed for the corneal front and back surface power, for the keratometric power, and for the total keratometry (IOLM) and real power (CASIA). The power vector components in 45°/135° were reversed in sign for all left eyes in our dataset in order to present the data in the same orientation as for right eyes. Mean, SD, Median, and 2.5% / 97.5% refer to the arithmetic mean, standard deviation, median, and the lower and upper bounds of the 95% confidence interval respectively.

N = 88, data in dpt	IOLM			CASIA preoperatively			CASIA postoperatively		
Front surface	IOLMa_EQ_	IOLMa_C0_	IOLMa_C45_	CASIAa_EQ_pre_	CASIAa_C0_pre_	CASIAa_C45_pre_	CASIAa_EQ_post_	CASIAa_C0_post_	CASIAa_C45_post_
Mean	48.5266	0.8859	-0.0397	48.6658	0.8114	0.0318	48.9763	1.0458	0.0013
SD	1.7750	1.7477	0.9862	1.7558	1.5887	0.8311	1.5810	1.6388	0.9220
Median	48.5409	1.2855	-0.0403	48.6534	1.0408	-0.0316	49.1399	1.0806	-0.0991
2.5%	45.2312	-2.4539	2.0319	45.3627	-2.1185	-1.8067	46.1795	-2.2171	-1.9311
97.5%	52.0043	4.6185	1.8349	52.2907	4.2665	1.7782	52.5110	4.5684	1.8890
Back surface	IOLMp_EQ_	IOLMp_C0_	IOLMp_C45_	CASIAp_EQ_pre_	CASIAp_C0_pre_	CASIAp_C45_pre_	CASIAp_EQ_post_	CASIAp_C0_post_	CASIAp_C45_post_
Mean	-5.7897	-0.3142	-0.0423	-6.1810	-0.3064	-0.0276	-6.2082	-0.3280	-0.0309
SD	0.2409	0.2085	0.1309	0.2506	0.2169	0.1313	0.2253	0.2162	0.1559
Median	-5.7991	-0.3211	-0.0556	-6.1754	-0.2942	-0.0194	-6.1925	-0.3168	-0.0265
2.5%	-6.3104	-0.7985	-0.2759	-6.7170	-0.8219	-0.2852	-6.7090	0.8558	0.3088
97.5%	-5.3338	0.0643	0.2224	-5.7225	0.0906	0.2281	-5.7785	0.0609	0.3095
keratometry	IOLMk_EQ_	IOLMk_C0_	IOLMk_C45_	CASIAk_EQ_pre_	CASIAk_C0_pre_	CASIAk_C45_pre_	CASIAk_EQ_post_	CASIAk_C0_post_	CASIAk_C45_post_
Mean	42.8479	0.7822	-0.0351	42.9709	0.7164	0.0280	43.2451	0.9234	0.0011
SD	1.5673	1.5432	0.8708	1.5503	1.4028	0.7338	1.3960	1.4471	0.8141
Median	42.8606	1.1351	-0.0356	42.9599	0.9190	-0.0279	43.3895	0.9541	-0.0875
2.5%	39.9382	-2.1667	-1.7941	40.0543	-1.8706	-1.5953	40.7755	-1.9576	-1.7051
97.5%	45.9187	4.0781	1.6202	46.1716	3.7672	1.5701	46.3652	4.0338	1.6680
TK/Real	IOLMt_EQ_	IOLMt_C0_	IOLMt_C45_	CASIAr_EQ_pre_	CASIAr_C0_pre_	CASIAr_C45_pre_	CASIAr_EQ_post_	CASIAr_C0_post_	CASIAr_C45_post_
Mean	42.8512	0.5794	-0.0809	42.6866	0.5187	0.0053	42.9000	0.7237	-0.0248
SD	1.5632	1.5709	0.8890	1.5516	1.4138	0.7321	1.3965	1.4688	0.7981
Median	42.8934	0.9465	-0.1169	42.6300	0.7227	-0.0199	42.9250	0.7353	-0.0966
2.5%	39.9858	-2.4624	-1.8521	39.8227	-2.0047	-1.5493	40.3700	-2.1991	-1.5914
97.5%	46.0026	3.7477	1.5624	45.9205	3.4703	1.5152	45.8350	3.7628	1.5009

**Fig 1:**
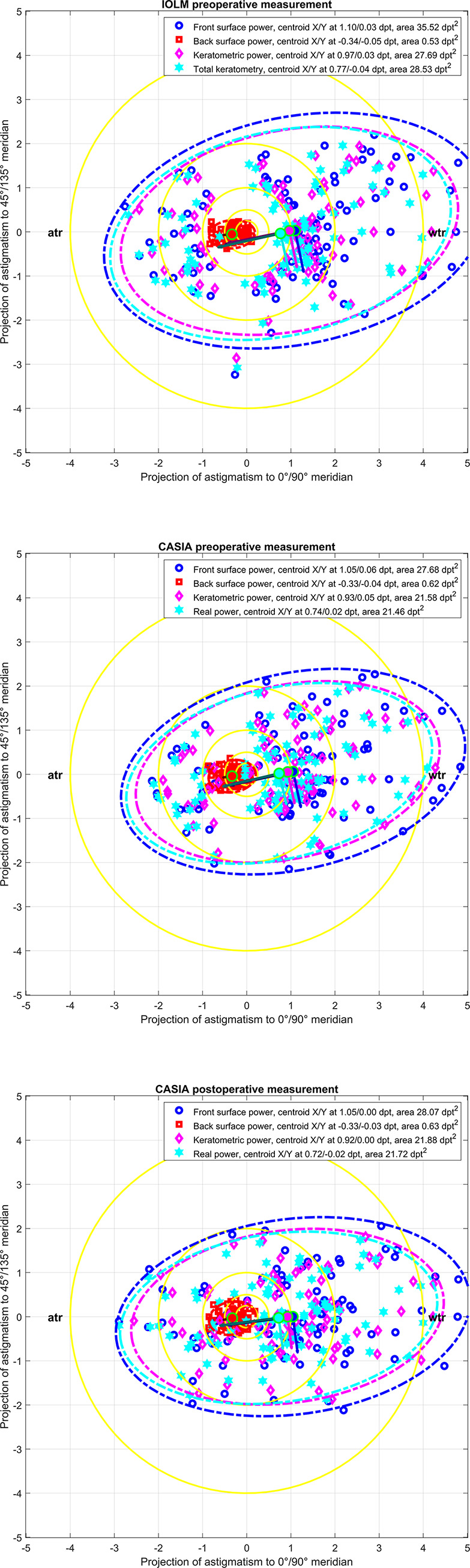
Double angle plots for the power vector components for the preoperative examination with the IOLM (upper graph), the preoperative examination with the CASIA (middle graph) and the postoperative examination with the CASIA (lower graph). The double angle plots overlay the power vector components C0 and C45 for the corneal front and back surface, the keratometric power, and the total keratometry (IOLM) / real power (CASIA) for the entire dataset (N = 88). The error ellipses (ellipse area mentioned in the labels) indicate the 90% confidence intervals and the centroids (filled dots, X / Y coordinates mentioned in the legend) together with the orientation of the ellipses (major and minor half axis indicated by dark and bright lines starting at the centroids). The yellow rings indicate astigmatism below 0.25, 0.5, and 1.0 dpt respectively. For left eyes, the C45 power vector components are reversed in sign in order to present the data in the same orientation as for right eyes. wtr refers to with-the-rule and atr to against-the-rule astigmatism.

**Fig 1** shows the double angle plots for the power vector components for the preoperative examination with the IOLM (upper graph), the preoperative examination with the CASIA (middle graph) and the postoperative examination with the CASIA (lower graph). The double angle plots overlay the power vector components C0 and C45 for the corneal front and back surface, the keratometric power, and the total keratometry (IOLM) / real power (CASIA) together with the centroids and the 90% error ellipses. We can clearly see from the graphs that the corneal front surface, the keratometry, and the total keratometry / real power show, on average, an astigmatism with the rule (with positive values of the C0 component), whereas the corneal back surface shows an astigmatism against the rule (with negative values of the C0 component).

**Table 2** lists the changes in power vector components for the entire dataset from the preoperative measurement with the IOLM and the CASIA to the postoperative measurement with the CASIA. From this table can see that the EQ component of the corneal power vector is systematically different for the preoperative measurement with the IOLM as compared to the postoperative measurement with the CASIA. The EQ front surface power and keratometric power are systematically higher with the CASIA measurement (on average 0.13 dpt and 0.12 dpt), whereas the respective vector component for the corneal back surface power and the real power are systematically lower compared to the back surface power and total keratometry of the IOLM (on average -0.26 and -0.21 dpt).

**Table 2:** Difference of the power vector components from the preoperative measurement with the IOLMaster 700 (IOLM) or the Casia2 (CASIA) to the postoperative measurement with the CASIA. Decompositions into power vectors were performed for the corneal front and back surface power, for the keratometric power, and for the total keratometry (IOLM) and real power (CASIA). The power vector components in 45°/135° were reversed in sign for all left eyes in our dataset in order to present the data in the same orientation as for right eyes. Mean, SD, Median, and 2.5% / 97.5% refer to the arithmetic mean, standard deviation, median, and the lower and upper bounds of the 95% confidence interval respectively.

N = 88, data in dpt	Difference CASIA postoperatively—IOLM			Difference CASIA postoperatively—preoperatively		
Front surface	CASIAa_EQ__post—IOLMa_EQ_	CASIAa_C0__post–IOLMa_C0_	CASIAa_C45__post–IOLMa_C45_	CASIAa_EQ__post—CASIAa_EQ__pre	CASIAa_C0__post—CASIAa_C0__pre	CASIAa_C45__post–CASIAa_C45__pre
Mean	0.1343	-0.0495	-0.0277	-0.0297	-0.0049	-0.0594
SD	0.3062	0.5316	0.4777	0.2341	0.3949	0.3180
Median	0.1112	-0.0003	-0.0030	-0.0303	0.0152	-0.0813
2.5%	-0.5631	-1.2111	-1.0019	-0.5044	-0.7934	-0.7520
97.5%	0.7454	0.8434	0.9310	0.4651	0.7530	0.5468
Back surface	CASIAp_EQ__post—IOLMp_EQ_	CASIAp_C0__post–IOLMp_C0_	CASIAp_C45__post–IOLMp_C45_	CASIAp_EQ__post—CASIAp_EQ__pre	CASIAp_C0__post—CASIAp_C0__pre	CASIAp_C45__post–CASIAp_C45__pre
Mean	-0.3582	0.0136	0.0269	0.0422	0.0032	0.0111
SD	0.0718	0.0920	0.0920	0.0678	0.0672	0.0712
Median	-0.3526	0.0021	0.0170	0.0441	-0.0003	0.0045
2.5%	-0.5198	-0.1808	-0.1264	-0.0944	-0.1224	-0.1264
97.5%	-0.2326	-0.2237	0.2625	0.1362	0.1598	0.1366
keratometry	CASIAk_EQ__post—IOLMk_EQ_	CASIAk_C0__post–IOLMk_C0_	CASIAk_C45__post–IOLMk_C45_	CASIAk_EQ__post—CASIAk_EQ__pre	CASIAk_C0__post—CASIAk_C0__pre	CASIAk_C45__post–CASIAk_C45__pre
Mean	0.1186	-0.0437	-0.0244	-0.0262	-0.0043	-0.0525
SD	0.2703	0.4694	0.4218	0.2067	0.3487	0.2808
Median	0.0982	-0.0003	-0.0027	-0.0268	0.0134	-0.0718
2.5%	-0.4972	-1.0693	-0.8847	-0.4453	-0.7006	-0.6640
97.5%	0.6582	0.7447	0.8221	0.4107	0.6649	0.4828
TK/Real	CASIAr_EQ__post—IOLMt_EQ_	CASIAr_C0__post–IOLMt_C0_	CASIAr_C45__post–IOLMt_C45_	CASIAr_EQ__post—CASIAr_EQ__pre	CASIAr_C0__post—CASIAr_C0__pre	CASIAr_C45__post–CASIAr_C45__pre
Mean	-0.2087	-0.0416	0.0114	-0.0607	-0.0116	-0.0452
SD	0.3047	0.5204	0.4536	0.2580	0.3953	0.3131
Median	-0.2237	-0.0040	0.0113	-0.0600	-0.0043	-0.0659
2.5%	-0.8347	-1.1070	-0.8797	-0.5080	-0.8194	-0.6730
97.5%	0.3756	0.8550	0.9595	0.5890	0.8007	0.5636

**Fig 2:**
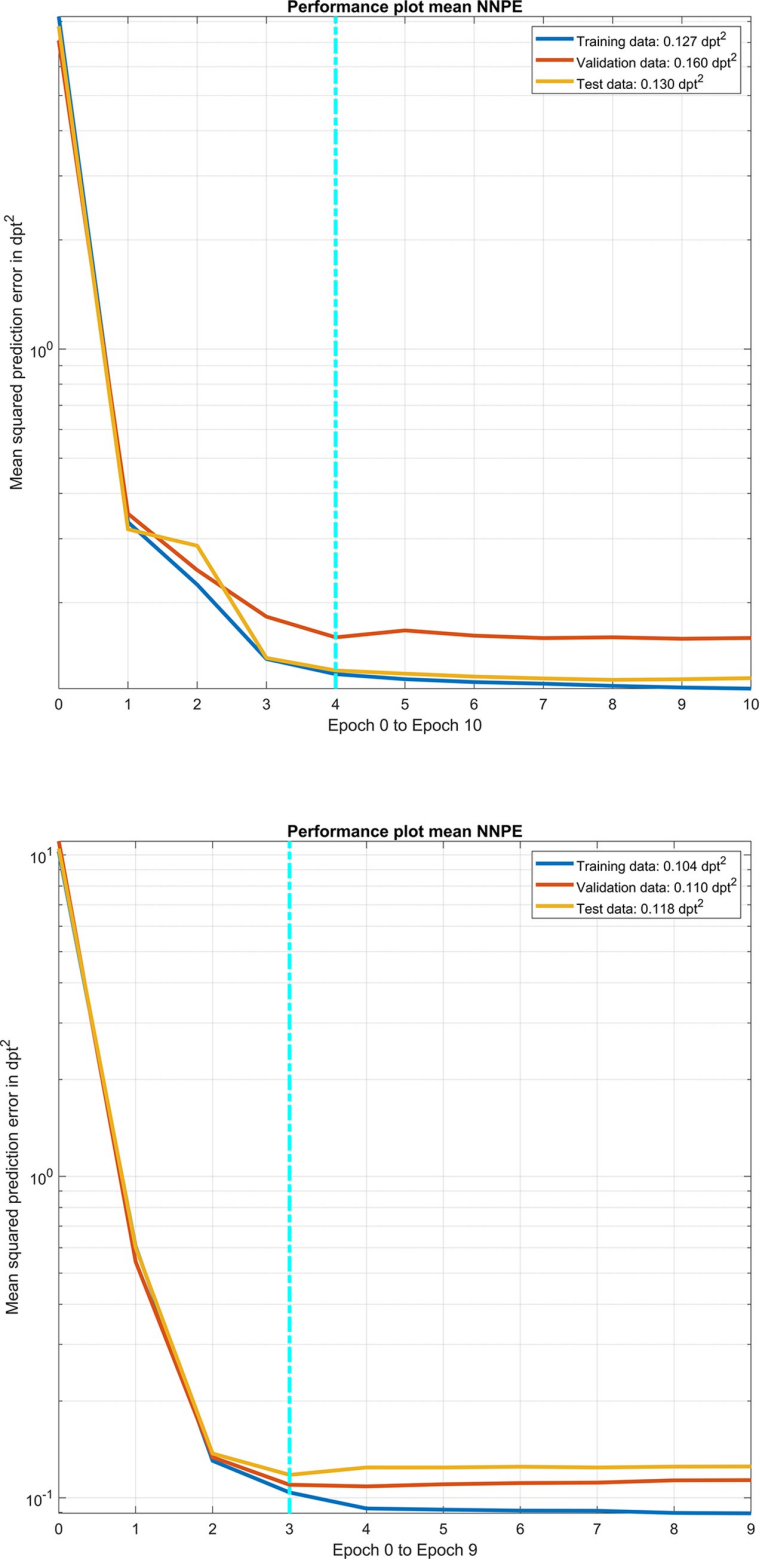
Performance plot of our final version of the feedforward neural network with 2 hidden layers and 10/8 neurons per layer with the mean squared prediction error of the network applied to the training data, validation data and test data for variations of iterations (epochs). Targeting to the power vector components of the postoperative CASIA real power measurement, the upper graph / lower graph shows the performance of the prediction made with the power vector components of the preoperative IOLM / CASIA keratometric power measurement. The mean squared prediction error for the training, validation, and test data are provided for the best epoch (iteration) indicated by the vertical dashed cyan line in the figure legend.

**Fig 2** shows the performance plot of our final version of the feedforward neural network with 2 hidden layers and 10/8 neurons per layer with the mean squared prediction error of the network applied to the training data, validation data and test data for variations of iterations (epochs). Targeting the power vector components of the postoperative CASIA real power measurement, the upper graph / lower graph shows the performance of the prediction made with the power vector components of the preoperative IOLM / CASIA keratometric power measurement. The mean squared prediction error for the training, validation, and test data are provided for the best epoch (f iterations for the neural network based on IOLM data and 3 iterations for the network based on CASIA data) and indicated with the vertical dashed cyan line in the figure legend.

The multivariate linear regression model mapping the preoperative power vector components (keratometric power) derived with the IOLM to the postoperative power vector components (real power) derived with the CASIA based on the training data is given for right (OD) and left (OS) eyes by:

$$\begin{array}{l} \mathrm{OD}:\;\left[\begin{array}{c} {\mathrm{REGCASIAr}}_{C0}\_\mathrm{post}\\ {\mathrm{REGCASIAr}}_{C45}\_\mathrm{post} \end{array}\right]=\left[\begin{array}{cc} 0.9150 & -0.0472\\ -0.0018 & 0.6952 \end{array}\right]\cdot \left[\begin{array}{l} IOLMk_{C0}\\ IOLMk_{C0} \end{array}\right]+\left[\begin{array}{l} -0.1823\\ -0.0229 \end{array}\right]\\ \mathrm{OS}:\;\left[\begin{array}{c} {\mathrm{REGCASIAr}}_{C0}\_\mathrm{post}\\ {\mathrm{REGCASIAr}}_{C45}\_\mathrm{post} \end{array}\right]=\left[\begin{array}{ll} 0.9150 & 0.0472\\ 0.0018 & 0.6952 \end{array}\right]\cdot \left[\begin{array}{l} IOLMk_{C0}\\ IOLMk_{C0} \end{array}\right]+\left[\begin{array}{c} -0.1823\\ 0.0229 \end{array}\right] \end{array}$$

With a logarithmic likelihood value of logL = -57.1781.

The respective multivariate linear regression model mapping the preoperative power vector components (keratometric power) derived with the CASIA to the postoperative power vector components (real power) derived with the CASIA based on the training data is given for right (OD) and left (OS) eyes by:

$$\begin{array}{ccc} \mathrm{OD}:\;\left[\begin{array}{c} {REGCASIAr}_{C0}\_post\\ {REGCASIAr}_{C45}\_post \end{array}\right] & = & \left[\begin{array}{cc} 1.0253 & -0.0023\\ -0.0473 & 0.8774 \end{array}\right]∙\left[\begin{array}{c} {CASIAk}_{C0}\_pre\\ {CASIAk}_{C0}\_pre \end{array}\right]+\left[\begin{array}{c} -0.2716\\ -0.2716 \end{array}\right]\\ \mathrm{OS}:\;\left[\begin{array}{c} {REGCASIAr}_{C0}\_post\\ {REGCASIAr}_{C45}\_post \end{array}\right] & = & \left[\begin{array}{cc} 1.0253 & 0.0023\\ 0.0473 & 0.8774 \end{array}\right]∙\left[\begin{array}{c} {CASIAk}_{C0}\_pre\\ {CASIAk}_{C0}\_pre \end{array}\right]+\left[\begin{array}{c} -0.2716\\ 0.2716 \end{array}\right] \end{array}$$

With a logarithmic likelihood value of logL = -37.8488.

In **Table 3** the fit error (difference between the C0 and C45 power vector components derived from the postoperatively measured CASIA real power and the respective components predicted by the feedforward shallow neural network or the multivariate linear regression) for the entire dataset (N = 88) is listed. The prediction from the feedforward neural network performs slightly better compared to the multivariate linear regression, and the prediction based on the preoperative CASIA data is slightly superior to the prediction based on the preoperative IOLM data. In the table, the arithmetic mean, standard deviation, and median fit error are listed together with the lower and upper bounds of the 95% confidence interval.

**Table 3:** Fit error (difference between the C0 and C45 power vector components derived from the postoperatively measured CASIA real power and the respective components predicted with the feedforward shallow neural network or the multivariate linear regression) for the entire dataset (N = 88). On the left side / right side the prediction error derived from the prediction models from the preoperative IOLM / CASIA keratometric data is shown. The prediction with the neural network performs slightly better as compared to the multivariate linear regression, and the prediction based on the preoperative CASIA data is slightly superior to the prediction based on the preoperative IOLM data. The power vector components in 45°/135° were reversed in sign for all left eyes in our dataset in order to present the data in the same orientation as for right eyes. Mean, SD, Median, and 2.5% / 97.5% refer to the arithmetic mean, standard deviation, median, and the lower and upper bounds of the 95% confidence interval respectively.

Fit error in dpt N = 88	Based on preoperative IOLMaster 700 (IOLM) keratometry data				Based on preoperative Casia2 (CASIA) keratometry data			
	Neural network		Linear regression		Neural network		Linear regression	
	CASIAr_C0__post- NNCASIAr_C0__post	CASIAr_C45__post- NNCASIAr_C45__post	CASIAr_C0__post- REGCASIAr_C0__post	CASIAr_C45__post- REGCASIAr_C45__post	CASIAr_C0__post- NNCASIAr_C0__post	CASIAr_C45__post- NNCASIAr_C45__post	CASIAr_C0__post- REGCASIAr_C0__post	CASIAr_C45__post- REGCASIAr_C45__post
Mean	-0.0333	-0.0170	0.0222	-0.0179	-0.0029	0.0219	0.0441	0.0073
SD	0.3686	0.2974	0.4568	0.3671	0.3376	0.2556	0.3820	0.2981
Median	-0.0111	-0.0410	0.0575	-0.0268	-0.0072	0.0083	0.0453	0.0105
2.5%	-0.8380	-0.6518	-0.9556	-0.8008	-0.7130	-0.4476	-0.7308	-0.6241
97.5%	0.6777	0.5468	0.9338	0.6515	0.6486	0.5348	0.8367	0.5634

**Fig 3:**
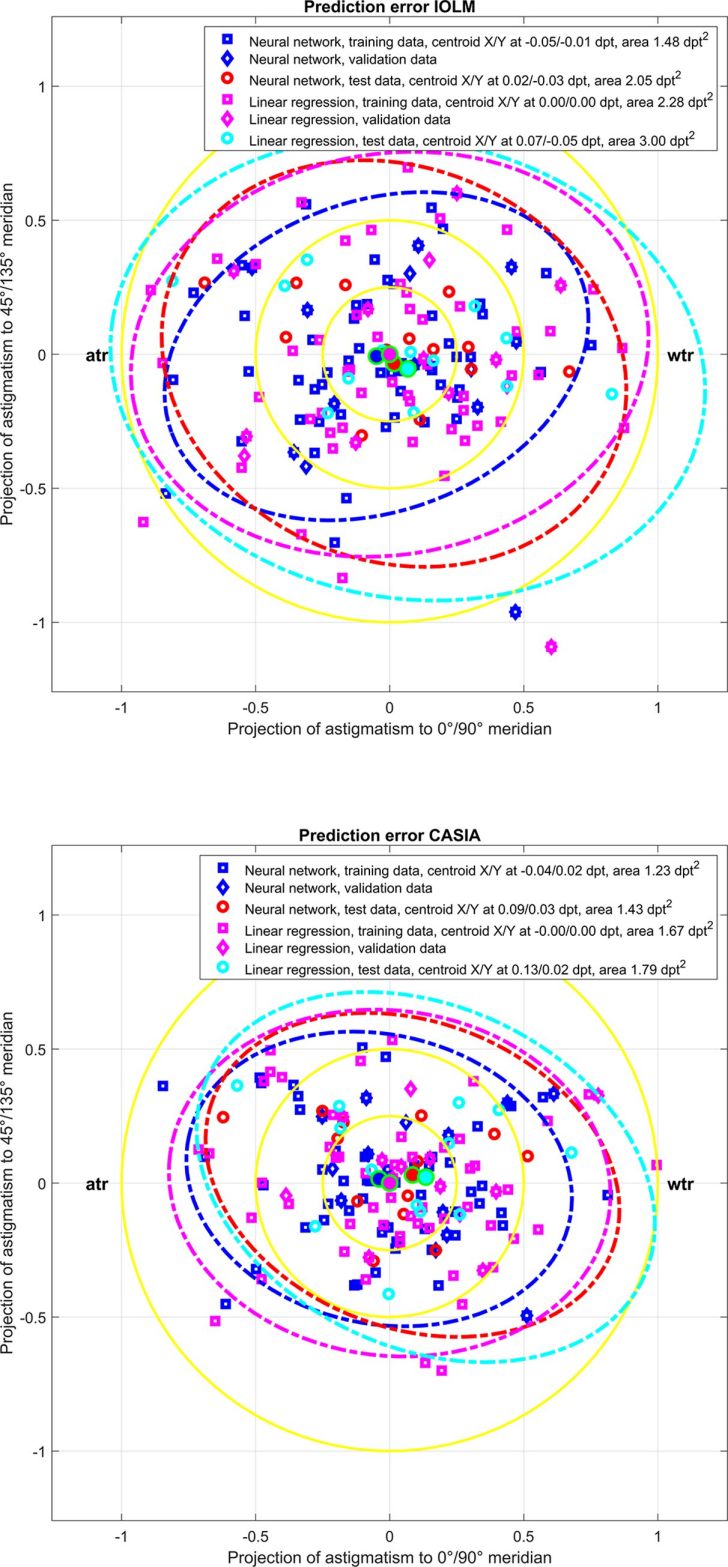
Double angle plots of prediction error (difference between the C0 and C45 power vector components derived from the postoperatively measured CASIA real power and the respective components predicted with the feedforward shallow neural network or the multivariate linear regression) for the entire dataset (N = 88). In the upper graph the prediction error is displayed for the situation that the feedforward neural network or the multivariate linear regression is based on the power vector components of the preoperative IOLM measurement (keratometric power). In the lower graph the prediction error is displayed for the situation that the feedforward neural network or the multivariate linear regression is based on the power vector components of the preoperative CASIA measurement (keratometric power). The graph shows the prediction error separately for the training data (N = 62), the validation data (N = 13), and test data (N = 13). The error ellipses (ellipse area mentioned in the labels) indicate the 90% confidence intervals and the centroids (filled dots, X / Y coordinates mentioned in the legend). The yellow rings indicate astigmatism below 0.25, 0.5, and 1.0 dpt respectively. For left eyes, the C45 power vector components are reversed in sign in order to present the data in the same orientation as for right eyes. ‘wtr’ refers to with-the-rule and ‘atr’ to against-the-rule astigmatism.

**Fig 3** shows the double angle plot of the prediction error data listed in **Table 3** for the entire dataset (N = 88). In the upper graph the prediction error is displayed for the situation where the feedforward neural network or the multivariate linear regression is based on the power vector components of the preoperative IOLM measurement (keratometric power). In the lower graph the prediction error is displayed for the situation where the feedforward neural network or the multivariate linear regression is based on the power vector components of the preoperative CASIA measurement (keratometric power). The graph shows the performance separately for the training data (N = 62), the validation data (N = 13), and test data (N = 13). From the error ellipses (shown for the training and test data only) we can see that the feedforward neural network performs slightly better (smaller error ellipses) compared to the multivariate linear regression. There is no systematic difference between the performance of the predictions applied to the training data or to the test data. The prediction performance is in the same range whether the preoperative keratometric power data from the IOLM or the CASIA are used to predict the postoperative real power data from the CASIA.

## Discussion

The largest challenge in biometry and intraocular lens power calculation is the prediction of the parameters of the pseudophakic eye from the preoperatively measured parameters of the phakic eye. Preoperatively we typically record distances such as the axial length, the phakic anterior chamber depth, the crystalline lens thickness or the central corneal thickness, together with the corneal anterior surface curvature in both cardinal meridians and the orientation of the astigmatism axis [2, 8]. The curvature data of the corneal back surface are only recorded in rare cases, even if some modern biometers such as the IOLMaster 700, the Anterion, or the Pentacam AXL offer this option [5–7, 10, 15]. The reason for ignoring corneal back surface data is that most classical and modern IOL power calculation schemes do not consider information on the corneal back surface. All of these parameters are potentially subject to change during cataract surgery, where the change in corneal curvature might have the largest effect in the prediction uncertainty for the pseudophakic eye dependent on the location and technique of cataract incision [13].

In the case of rotationally symmetric IOLs we could restrict calculations to the average corneal power. This is mostly reflected by the mean value of the flat and steep meridian of manual or automated keratometry or the respective values from simulated keratometry of a topographer or tomographer. However, with tIOL power calculation it is mandatory to assess the astigmatism of the cornea and the change from the preoperative to the postoperative situation (the surgically induced astigmatism SIA) in addition to considering the equivalent power of the cornea. Many papers have been published with vector analyses focusing on the effect of astigmatic changes resulting from cataract surgery, mostly caused by the incision locations and techniques [1, 16–24]. As keratometers are restricted to measurement of the corneal front surface curvature, they use a keratometer to convert the corneal radius of curvature derived in both meridians into the ‘keratometric power’ [8]. However, it is well-known in ophthalmology (Javal’s rule), that the contribution of the back surface astigmatism to the total astigmatism of the cornea is not well reflected by a keratometer, because the corneal back surface typically shows more astigmatism against the rule than keratometers indicate [9, 10, 21].

Several statistical prediction strategies have been developed for including this information into tIOL calculations. These should map the corneal back surface contribution where a direct corneal back surface measurement is not available or if the tIOL calculation scheme requires keratometric data [8].

In the present study we selected eyes where a tIOL has been implanted to correct for corneal astigmatism from a cataractous population. All eyes showed preoperative corneal keratometric astigmatism of at least 1.0 dpt derived with the IOLMaster 700 biometer (including the corneal back surface and total keratometry measurement feature). In addition to biometric measurement, the Casia2 anterior segment tomographer was used preoperatively and postoperatively to analyse the corneal front and back surface and the real power which yields the composite of the corneal front and back surface measurement. The standard notation familiar to all ophthalmologists is not useful to assess corneal power, therefore we decided to transform all corneal radius or power data into power vector components. These components can be algebraically added or subtracted to consider the effect of refractive surfaces or to calculate differences between instruments or the change between different time points [5, 6].

For all 3 measurements (the preoperative measurement with the IOLM and the CASIA and postoperative measurement with the CASIA) we recorded the corneal front surface, back surface, keratometric, and total keratometry / real power data. For a simple estimation based on the Liou-Brennan schematic model eye, and assuming a corneal front surface radius of 7.77 mm and a fixed front-to-back surface radius ratio of 7.77/6.4 (together with a refractive index of cornea/aqueous humour of 1.376/1.336), then the corneal front / back surface power reads 48.3912 / -6.25 dpt (ratio -7.7426). For a 0.1 mm radii difference between both meridians in the corneal front surface obtain a front / back surface astigmatism of 0.6228 / 0.0804 dpt (perpendicular to the front surface astigmatism, ratio again -7.7426). Comparing this data to the preoperative or postoperative measurement with the IOLM or the CASIA as listed in **Table 1**, we see that the IOLM systematically underestimates the (negative) back surface power (mean ratio -8.3815 for the IOLM in contrast to -7.8735 / -7.8890 for the CASIA preoperatively / postoperatively). However, we also see from the centroids for the corneal front and back surface astigmatism shown in **Fig 1** that the corneal back surface astigmatism contribution is much more than 1/7.7426 of the front surface astigmatism as indicated by the schematic model eye [17]. This means that a keratometric measurement (restricted to the corneal front surface measurement) is not appropriate to determine the amount (and axis) of corneal astigmatism, even though very good refractive results are obtained after cataract surgery with proper formula constant optimisation.

This is mostly due to the fact that keratometers implicitly work with a fixed front to back surface curvature ratio. However, the corneal back surface is typically much steeper vertically than horizontally, which could reduce the total astigmatism (if the corneal front surface shows with the rule astigmatism), increase astigmatism (if the corneal front surface shows against the rule astigmatism) or even rotate the astigmatic axis (with oblique astigmatism at the front surface). In this study we wanted to analyse corneal astigmatism in detail and define prediction models based on keratometric measurement from the IOLM or CASIA intended to map the postoperative total corneal astigmatism for a temporal corneo-scleral 2.2 mm incision. We implemented 2 options of prediction models, one based on a shallow feedforward neural network and the second based on a robust multivariate linear regression model. After splitting our data randomly 70% / 15% / 15% into training, validation and test sets we used the training set for training and optimising the prediction and the test set for performance checking and validation [8, 9]. The validation set was used for back-propagation with the neural network. In a final step we used these prediction models to estimate the postoperative astigmatic power vector components C0 and C45 for the entire dataset, in order to get some insight in the characteristics of the prediction error. From **Fig 3** we can see that the prediction error of the total corneal astigmatism is mostly in a range of 1 dpt, and the centroids are pretty much centred indicating that there is no systematic offset (in the training, validation, and test set). It is obvious that the prediction model based on the preoperative CASIA data performs slightly better (smaller error ellipses) as compared to the prediction model based on the preoperative IOLM data. If we use the size of the 90% error ellipses as a measure for the uncertainty of the corneal astigmatism prediction, we have to be aware that based on the corneal front surface measurement with a biometer or a tomographer the precision of total corneal astigmatism prediction is within a range of 0.5–0.6 dpt with no clear preference for any astigmatism axis.

We should also keep in mind that after decomposition of the corneal power into power vector components we subsequently reversed the C45 vector component in sign for left eyes in order to consider all eyes as ‘right eyes’ [18]. While it is known from clinical experience that the astigmatic axes of both eyes of an individual are quite often ‘mirrored’ we have not found a final statistical proof of this finding in the literature [25]. However, reversing the sign of the C45 vector component for left eyes means that in the multivariate linear regression models as shown in the Results section we have to use the prediction ‘case sensitive’ for left and right eyes [25]. Nevertheless, as the 2^nd^ intercept element and both off-diagonal elements in the (2x2) matrix (to be reversed for left eyes) are close to 0 the effect of not reversing the sign of the C45 components is in general expected to be low.

As a rule of thumb, if we wish to estimate the keratometric / real power of the CASIA after cataract surgery with implantation of a tIOL from the respective preoperative IOLM keratometric / total keratometry values, we have to add on average 0.12 dpt / subtract on average 0.21 dpt to the equivalent power of the cornea (**Table 2**), whereas the systematic differences of the astigmatic vector components C0 and C45 both are negligible (-0.04 and -0.02 dpt / -0.04 and -0.01 dpt). Furthermore, if we wish to estimate the change in keratometric / real power of the CASIA due to cataract surgery with implantation of a tIOL (postoperative minus preoperative), we noticed a clinically insignificant decrease in the equivalent power (-0.03 dpt / -0.06 dpt) and in both astigmatic vector components (0.00 and -0.05 dpt / -0.01 and -0.05 dpt). However, this correction of the systematic shift does not consider individual differences or changes which are up to ±1.1 dpt according to the limits of the 95% confidence intervals.

However, our study has several limitations: First, our data are based on a single measurement made preoperatively with the IOLMaster and pre- and postoperatively with the Casia2. Variations in the results due to multiple measurements [5–8] have not been considered in our prediction models. Second, the study population is relatively small with N = 88 eyes, and therefore a refinement of this prediction algorithm with a larger population is recommended. Third, we assumed a symmetry of left and right eyes in order to restrict the study to a single prediction model for left and right eyes. Similar symmetry assumptions have been used with other prediction algorithms to map keratometric astigmatism to total corneal astigmatism (e.g. the Abulafia-Koch correction [26]).

In **conclusion**, this paper shows that it may be difficult to predict the total corneal power of the pseudophakic eye to be used for the lens power calculation concept based on the biometric measurement of eyes before cataract surgery. For calculation of toric intraocular lenses, the astigmatism of the pseudophakic eye is required in addition to the average corneal power. Between different measurement modalities there could be systematic differences as shown for the IOLMaster and the Casia2, but even if there are no systematic changes from the preoperative to the postoperative situation both measured with the same device, there is a large variation both in the equivalent power and in both astigmatic power vector components following cataract surgery. With the IOLMaster the (negative) back surface power is typically underestimated. For toric lens power calculation, clinicians have to be aware that measurements restricted to the corneal front surface (manual or automated keratometers or topographers) use a keratometer index for converting front surface radii into keratometric power, and the effect of back surface astigmatism is not appropriately considered.
